# Immune Responses Induced by Recombinant *Bacillus Subtilis* Expressing the Hemagglutinin Protein of H5N1 in chickens

**DOI:** 10.1038/srep38403

**Published:** 2016-12-16

**Authors:** Chunxiao Mou, Liqi Zhu, Jingjing Yang, Wenwen Xu, Xiaoying Cheng, Qian Yang

**Affiliations:** 1 College of Veterinary Medicine, Nanjing Agricultural University, Weigang 1, Nanjing, Jiangsu, 210095, PR China

## Abstract

To develop an effective, safe, and convenient vaccine for the prevention of highly pathogenic avian influenza (HPAI) H5N1, we have constructed a recombinant *Bacillus subtilis* strain (*B.S.-HA*) expressing the hemagglutinin (HA) protein. Then we evaluated the immune function in chicken bone marrow derived dendritic cells (BM-DCs), and the immune response after oral immunization. Our results show that the recombinant *Bacillus subtilis B.S.-HA* could be sampled by BM-DCs *in vitro* and increase the BM-DCs major histocompatibility complex (MHC) II phenotype. The weight, height of the small intestine villus, and lymphoid tissue area of the ileum increased significantly in *B.S.-HA* immunized chickens (*P* < 0.05 or *P* < 0.01). *B.S.-HA* induced the secretion of cytokines and the expression of Toll-like receptors in the trachea and small intestine (*P* < 0.05 or *P* < 0.01). In addition, *B.S.-HA* elevated the specific IgA titers in the trachea, IgG and HI antibody titers in serum (*P* < 0.05 or *P* < 0.01). Therefore, *B.S.-HA* provides a potential novel strategy and approach for developing an H5N1 vaccine.

The highly pathogenic avian influenza (HPAI) virus, H5N1, is a worldwide threat to the poultry industry. The virus evolved rapidly and shows high genetic diversity, a broad host range, and ongoing circulation in birds. H5N1 also has the potential to cause a human pandemic^1^. The current vaccination (intramuscular immunization) against HPAI is associated with reduced morbidity and mortality in poultry. However, intramuscular injection can inhibit growth and fail to induce sufficient mucosal immunity^2^. Many investigators are pursuing more convenient and economical routes to construct new vaccines, such as recombinant subunit vaccines using baculovirus^3^, plasmid DNA^4^, or replication-incompetent adenoviral vectors^5^. H5N1 infects animals mainly through their respiratory and intestinal tracts^6^. Protective H5N1 antigens may induce an effective mucosal immune response to prevent invasion by H5N1, as many mucosa-associated lymphoid tissues underneath the epithelia of the respiratory and intestinal tracts. Therefore, developing a mucosal vaccine is a feasible strategy.

*Bacillus subtilis* is a nonpathogenic Gram-positive bacterium, a novel probiotic and biocontrol bacterium. *B. subtilis* offers unique resistance properties and survives under extreme conditions, such as extreme temperatures, desiccation, and exposure to noxious chemicals^7^. *B. subtilis* is widely used as a vehicle for heterologous antigen expression and protective immunization^8^,^9^. In addition, our previous study demonstrated that *B. subtilis* remarkably improved the immunoprotective efficacy of whole inactivated H5N1 influenza virus via oral immunization in chickens by enhancing local and systemic immune responses^10^. *B. subtilis* can also induce non-specific immune response against infection, increase IgA production, and regulate the balance of the Th1 and Th2 pathways^11^.

In this study, a recombinant *B. subtilis* strain (*B.S.-HA*) expressing hemagglutinin (HA) protein was constructed, and the efficacy of oral immunization of *B.S.-HA* was evaluated in chickens. *B.S.-HA* promoted growth, and induced cytokine secretion and expression of Toll-like receptors (TLRs). In addition, *B.S.-HA* could be sampled by chicken bone marrow-derived dendritic cells (BM-DCs), and it induced the expression of major histocompatibility complex (MHC) II.

## Materials and Methods

### Bacterial strains, plasmids, virus and animals

*B. subtilis* WB800N, and *pHT43* plasmid were kindly provided by Dr. Xuewen Gao. *pMD-19T-H5N1* plasmid was kindly provided by the Jiangsu Academy of Agricultural Sciences. The inactivated avian influenza virus (IAIV) H5N1 was kindly provided by Qingdao Municipal Center for Disease Control & Prevention. Specific-pathogen-free (SPF) chickens (Hyline) were kindly provided by Jiangsu Academy of Agricultural Sciences (Nanjing, China). The animal studies were approved by the Institutional Animal Care and Use Committee of Nanjing Agricultural University and followed the National Institutes of Health’s guidelines for the performance of animal experiments.

### Construction of recombinant *B. subtilis* strains

To obtain recombinant *B. subtilis* WB800N, a recombinant plasmid was constructed. Firstly, the HA fragments were amplified with primers F1/R1 (Table. 1) from *pMD-19T-H5N1* plasmid, *pHT43* plasmid was digested by restriction enzyme *BamH*I and *Sma*I. Secondly, the fragment was purified and inserted into vector *pHT43* plasmid by T4 DNA ligase (Thermo Scientific) to generate the vector *pHT43-HA*. The plasmid *pHT43-HA* was confirmed by restriction enzyme digestion with *BamH*I and *Sma*I. Finally, *pHT43* and *pHT43-HA* were transformed into *B. subtilis* WB800N, respectively, by electroporation as previously described^12^, the recombinant *B. subtilis* WB800N strains were named *B.S.* and *B.S.-HA*, respectively.

### Analysis of fusion protein

*B.S.* and *B.S.-HA* were grown in LB medium with 5 μg/ml chloramphenicol, adding IPTG (0.1 M) at growth phase (OD_600_ = 0.5), then grown for 3 h. The bacterial were washed three times with sterile phosphate-buffered saline (PBS) and collected. Then the *B.S.* and *B.S.-HA* was ultrasonicated. For immunodetection of the fusion proteins by western blotting^13^, mouse anti-HA (Abcam, USA), followed by HRP-conjugated goat anti-mouse IgG (Sigma, USA) were used. Then the blots were developed by enhanced chemiluminescence.

### Isolation and culture of chicken bone marrow derived dendritic cells (BM-DCs)

Chicken BM-DCs were generated as previous method^14^. Femurs and tibias of 4–6 week-old chickens were removed and isolated from the surrounding muscle tissue using sterile instruments. The bones were washed three times with 0.01 M PBS, and then both ends of the bones were cut with scissors in the dish. The marrow was flushed with 0.01 M PBS using a 10 ml syringe with a 0.45-mm-diameter needle. Clusters within the marrow suspension were disaggregated by vigorous pipetting. After one wash in PBS, the cells were suspended in 0.01 M PBS and loaded onto an equal volume of Histopaque-1119 (Sigma-Aldrich, UK) and centrifuged at 2500 rpm for 25 min. Cells at the interface were collected and washed twice with 0.01 M PBS.

Cells obtained from femurs and tibias were cultured at a final concentration of 2 × 10^6^ cells/ml in six-well plates in the culture medium containing RPMI-1640 (Gibco, USA), 10% fetal bovine serum (FBS) (Wisent, CAN), 50 ng/ml recombinant chicken GM-CSF (Abcam, USA), 10 ng/ml IL-4 (Kingfisher, USA), 1 U/ml penicillin and 1 μg/ml streptomycin, for 7 days at 37 °C and 5% CO_2_. Half of the medium was replaced with fresh complete medium at day 2 and day 4 to remove non-adherent cells (such as dead cells and granulocytes). Effects of the recombinant chicken GM-CSF and IL-4 on cell differentiation were recorded by observing cell morphology, clustering and cell growth. The cell cultures were photographed during 7 days of culture with a digital camera on an inverted microscope.

### Purity and uptake phenotype assay

The chicken BM-DCs were stimulated with 0.01 M PBS, LPS (100 ng/ml), *B.S.* (10^7^ cfu/well) and *B.S.-HA* (10^7^ cfu/well) at 37 °C and 5% CO_2_ at day 6. After 24 h, cells were harvested by gentle pipetting and washed with 0.01 M PBS. Then, the immature BM-DCs (0.5 × 10^6^ cells/ml) were stained with 0.05 mg/ml of PE-conjugated mouse anti-human CD11c antibody (eBioscience, USA) and incubated for 20 min at room temperature. The purity of BM-DCs was evaluated based on the relative levels of CD11c expression. In addition, the mature BM-DCs were stained with 0.5 mg/ml of FITC-labeled mouse anti-chicken MHC II antibody (Abcam, USA) and then incubated for 20 min at room temperature, following washed with 0.01 M PBS and centrifuged at 1500 rpm for 5 min. DCs uptake was determined based on the relative levels of MHC II expression. After the final wash, cells were analyzed on a FACS Calibur (BD Bioscience, Cowley, UK).

### Antigen uptake assay

The chicken BM-DCs (0.5 × 10^6^ cells/ml) were incubated with DyLight 488-*B.S.* or DyLight 488-*B.S.-HA* (10^7^ or 10^8^ cfu/well) at 37 °C for 2 h or 3 h. The DCs were washed three times with PBS and then were analyzed by FACS.

### Immunization Schedule

The immunization schedule was described by other researchers^10^. In briefly, 10-day-old Hyline chickens were randomly divided into 4 groups of 30 chickens each and immunized. Nonimmunized control (PBS) chickens received oral administration of 150 μl of 0.01 M PBS. Groups of chickens were immunized orally with one of the following: 10^10^ cfu/kg *B.S.*, 10^10^ cfu/kg *B.S.-HA*, or 2 ml/kg IAIV. All chickens were immunized again one week after the first immunization. The body weights were detected from 6 chickens in each group every week.

### Collection of Samples

Blood samples were taken weekly from 6 chickens in each group after the first immunization and allowed to clot overnight at room temperature before serum was collected. Serum was separated by centrifugation and stored at −20 °C for detection of specific IgG. The chickens were killed 1, 3, 5, and 7 weeks after the first immunization. Trachea and small intestine tissue samples were taken from 6 chickens in each group, and washed with 0.5 ml of 0.01 M PBS repeatedly. The suspensions were centrifuged at 5,000 × g for 10 min, collected, and stored at −20 °C for specific IgA detection. The same location of the small intestine and ileum tissues from 6 chickens after 7 week immunization were fixed with Bonn’s liquid. Chickens were killed at 3 days after the second immunization, trachea and small intestine tissues and their mucosal suspensions were also collected. The mucosal suspensions were stored at −20 °C for detection of cytokines, and tissues stored in liquid nitrogen for TLRs expression by real-time quantitative PCR (RT-qPCR).

### Hematoxylin-eosin staining assay

The same location of small intestine and ileum tissues were fixed with Bonn’s liquid, embedded in paraffin and sectioned at 4 μm thickness. Hematoxylin-eosin staining was applied to the paraffin sections^15^. Then visualized by OLYMPUS CX23. The height of small intestine villi in the same location of the small intestine and the lymph tissue area of ileum at ten different fields in each chicken were counted for the statistical analysis.

### RNA isolation and quantitative RT-qPCR assay

Total RNA was extracted from trachea and small intestine tissues using a RNA extraction kit (Thermo Scientific) and subjected to reverse transcription with Prime Script RT-qPCR Kit (Takara, Dalian, CA), using an ABI 7500 instrument (Life Technologies)^16^. The data were reported as values normalized to a housekeeping gene (β-actin) to account for repeated measures. Specific primers were shown in Table 1.

### Enzyme-linked immunosorbent assay

Specific IgG in serum and IgA in mucosal suspensions were determined by enzyme-linked immunosorbent assay (ELISA) as previously described^17^. In brief, ELISA plates were coated with the purified recombinant HA protein (our laboratory saved) in carbonate buffer (pH = 9.6) at 4 °C overnight. Plates were saturated with PBS containing 1% BSA at 37 °C for 2 h. The serum and mucosal suspensions of dilution degrees were added and incubated for 1 h at 37 °C. Subsequently, the HRP-conjugated goat anti-chicken IgG and goat anti-chicken IgA (BETHYL, CA) were added to the wells and incubated for an additional 1 h at 37 °C. Subsequently, a substrate solution containing o-phenylenediamine (OPD) and H_2_O_2_ was added, the reaction was allowed to proceed for 15 min at room temperature before it was terminated by the stop solution. Finally, the absorbance was measured at 450 nm, using an automated ELISA reader (Molecular Devices, Shanghai, CA).

### Hemagglutination inhibition assay

To explore whether chicken generated avian influenza virus (AIV) neutralizing antibodies, the neutralization activity of serum antibody was determined by hemagglutination inhibition (HI) assays according to the Chinese National Standard of diagnostic techniques for highly pathogenic avian influenza and the Office International des Epizooties (OIE) standards (OIE, 2005).

### T-cell proliferation assays

The splenic lymphocyte were isolated from the all group chicken spleens after 7 week immunization, and then labelled with CFSE (Invitrogen, USA). Cells were cultured in a lymphocyte culture medium of 2 × 10^5^ cells per well in 24-well culture plates and stimulated by the purified recombinant HA protein (final concentration 10 μg/ml) for 72 h at 37 °C and 5% CO_2_. Cell proliferation assays were detected by FACS. Non-stimulated cells were used as negative controls.

### Statistical analysis

The results were expressed as the means ± the standard deviations (SD). Statistical significance was determined by oneway analysis of variance (ANOVA) followed by Dunnett’s t test to evaluate variations between groups; a *P* value of <0.05 was considered statistically significant.

## Results

### The construction of recombance plasmids and the analysis of fusion protein expression

The HA fragment (1702 bp) was inserted into the *pHT43* vector to generate the *pHT43-HA* shuttle vector (supplementary material Fig. 1A) and express HA in *B. subtilis* WB800N. We confirmed the presence of *pHT43-HA* by restriction enzyme digestion with *BamH*I and *Sma*I (supplementary material Fig. 1B). The *pHT43* or *pHT43-HA* plasmid was transformed into *B. subtilis* WB800N by electroporation, and the HA protein was immunodetected using an HA antibody. As shown in supplementary material Fig. 1C, the lane 1 and 2 were the *pHT43* plasmid group *B.S.*, the lane 3, 4, 5, and 6 were the recombinant *pHT43-HA* plasmid group *B.S.-HA*. Immunodetection revealed a clear positive band at 63 KDa in the recombinant *pHT43-HA* plasmid group *B.S.-HA*. The results show that the HA protein was successfully expressed in *B. subtilis* WB800N.

### The culture of chicken bone marrow derived dendritic cells (BM-DCs) *in vitro*

**Figure 1:**
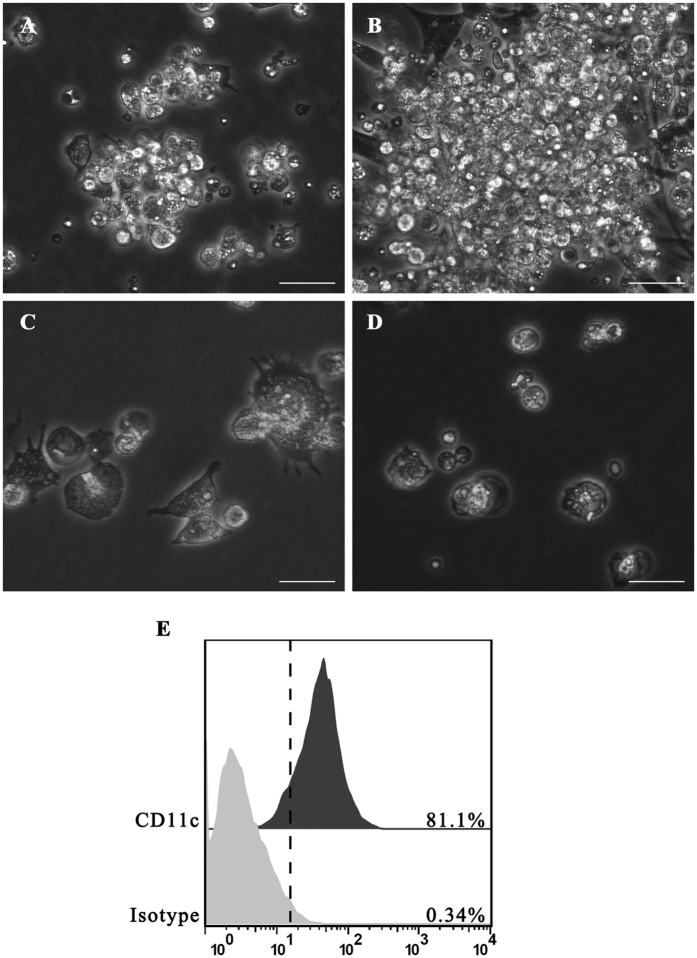
Morphology of chicken BM-DCs cultured with recombinant chicken GM-CSF and IL-4. (**A**) Light microscopy showed cell aggregates after 4 days of culture (100×). (**B**) The size of these aggregates increased after 24 h of stimulation with *B.S.* or *B.S.-HA* (100×). (**C**) Morphology of cells after 7 days of culture after stimulation with *B.S.* or *B.S.-HA* (400×). (**D**) Culture of chicken bone marrow cells without chicken GM-CSF and IL-4 (100×). Scale bars =20 μm. (**E**) More than 81% of immature chicken BM-DCs were CD11c^+^ by flow cytometry.

When suspensions of chicken bone marrow cells were cultured in the presence of granulocyte-macrophage colony-stimulating factor (GM-CSF) and interleukin (IL-)-4, the immature BM-DCs aggregates had attached to a layer of adherent cells by day 4 (Fig. 1A). These aggregates grew in size and were found to be floating or loosely adherent on day 7 (Fig. 1B), following *B.S., B.S.-HA*, and positive lipopolysaccharide (LPS) stimulation. Many individual cells and peripheral aggregates displayed a large veiled or dendritic appearance indicating maturation (Fig. 1C). When chicken bone marrow cells were cultured under the same conditions without GM-CSF or IL-4, no cell aggregates were observed, and only a few live cells remained in the plates by day 7 (Fig. 1D). Flow cytometry analysis of the cultured cells harvested on culture day 7 is shown in Fig. 1E. The immature DCs expressed high levels of cell surface and putative CD11c molecules.

### The improvement of the BM-DCs uptake

**Figure 2:**
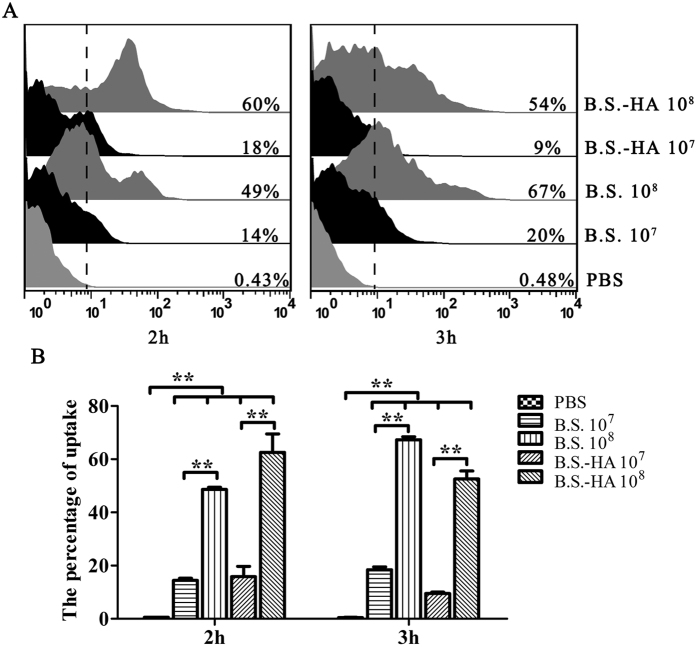
The analysis of *B. subtilis* sampled by BM-DCs. (**A**) The chicken BM-DCs (0.5 × 10^6^ cells/ml) were incubated with DyLight 488-*B.S.* or DyLight 488-*B.S.-HA* (10^7^ or 10^8^ cfu/well) at 37 °C for 2 h or 3 h, and analyzed by flow cytometry. (**B**) The data is displayed in the histogram. Data shown are the means ± s.d. of three samples. *P < 0.05; **P < 0.01. The error bars represent standard deviations.

**Figure 3:**
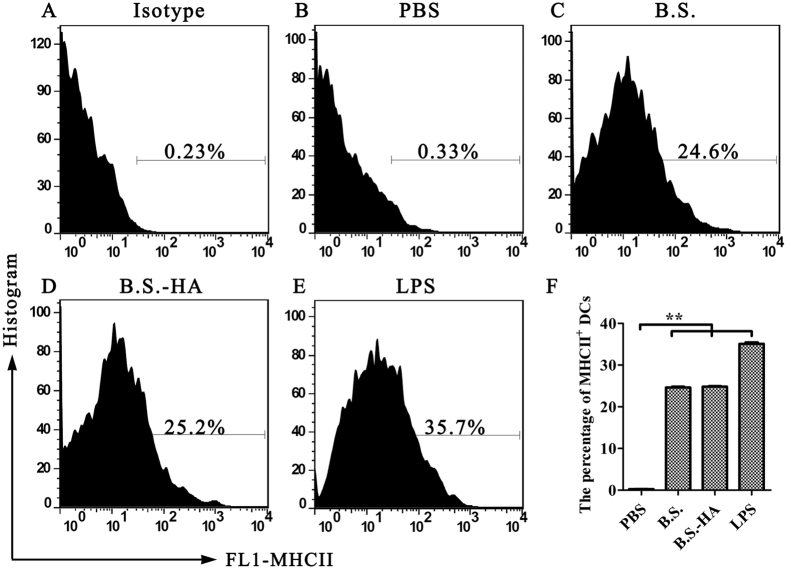
The analysis of MHC II phenotype expression in BM-DCs. (**A–E**) The chicken BM-DCs were stimulated with PBS, LPS (100 ng/ml), *B.S.* (10^7^ cfu/well) and *B.S.-HA* (10^7^ cfu/well) for 24 h, stained with 0.5 mg/ml of FITC-labeled mouse anti-chicken MHCII antibody, and analyzed by flow cytometry. (**F**) The data is displayed in the histogram. Data shown are the means ± s.d. of three samples. *P < 0.05; **P < 0.01. The error bars represent standard deviations.

Uptake is an important function of DCs. To explore whether *B.S.-HA* improves uptake by BM-DCs, different doses of DyLight 488 labelled *B.S.* or *B.S.-HA* were incubated with BM-DCs at 37 °C for 2 or 3 h, and the percentage of BM-DCs containing DyLight 488-*B.S.* or DyLight 488-*B.S.-HA* was detected by flow cytometry (Fig. 2A). The percentage of BM-DCs (Fig. 2B) containing DyLight 488-*B.S.* or DyLight 488-*B.S.-HA* increased (*P* < 0.01) with the dose of *B.S.* or *B.S.-HA*. The positive ratio of DCs exhibiting DyLight 488 fluorescence increased in the *B.S.* group, but decreased in the *B.S.-HA* group with the treatment time. In addition, expression of the chicken BM-DCs MHC II phenotype increased significantly after stimulated with *B.S.* or *B.S.-HA* (Fig. 3A-F) (P < 0.01).

### The changes of body weight, small intestinal villi height, and lymphoid tissue area

**Figure 4:**
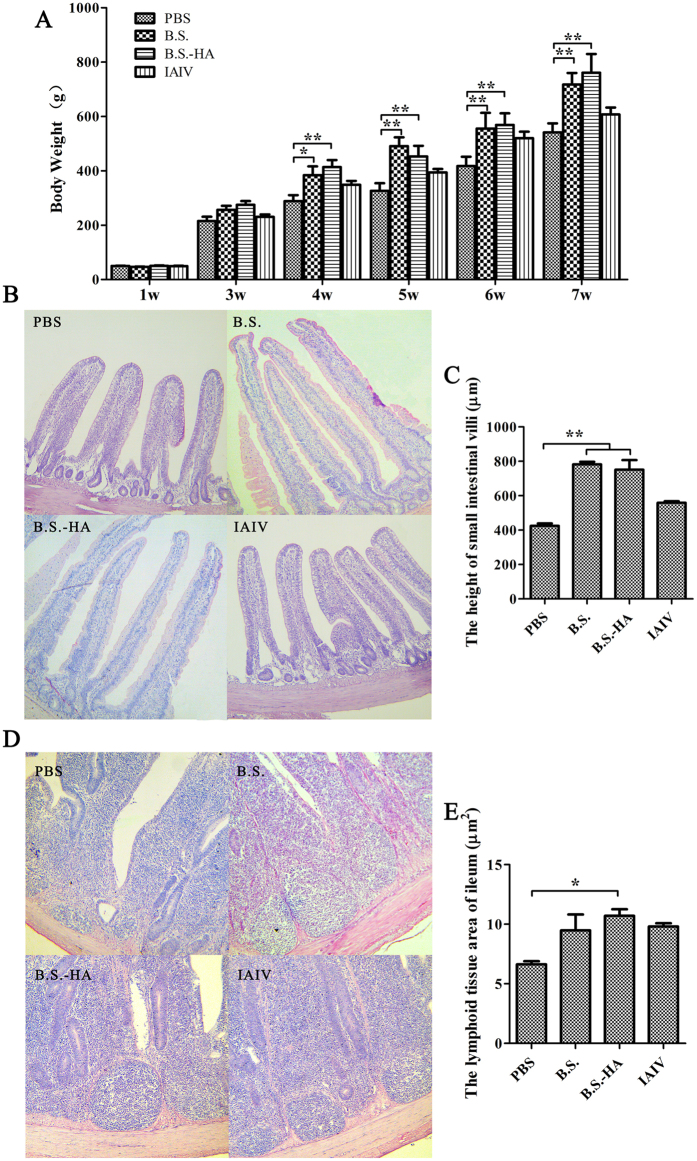
Changes of body weight, small intestinal villi height and lymphoid tissue area. (**A**) The body weights were counted in each group every week. (**B**) Hematoxylin-eosin stained on the same part of the small intestine and detected the intestinal villi height after immunized 7 weeks (100×). (**C**) The data is displayed in the histogram. (**D**) Hematoxylin-eosin stained on ileum and detected the lymphoid tissue area after immunized 7 weeks (400×). (**E**) The data is displayed in the histogram. Data shown are the means ± s.d. of six samples. *P < 0.05; **P < 0.01. The error bars represent standard deviations.

*B. subtilis* has been used as a feed additive to improve the microorganismal environment in the intestine^18^. We weighed the chickens after they developed immunity (Fig. 4). After stimulation with *B.S.* or *B.S.-HA*, a significant increase in the weight of chickens was observed (*P* < 0.01 or *P* < 0.05), compared with the other groups. Due to the important role of intestinal villi in nutrient absorption, we explored whether the intestinal villi were altered in chickens fed *B.S.* or *B.S.-HA*. As shown in Fig. 4B and D, paraffin sections from the same location of the small intestine in both groups was stained with hematoxylin-eosin, and the heights of the intestinal villi were counted 7 weeks after immunization (Fig. 4C and E). After stimulation with *B.S.* or *B.S.-HA*, a significant increase in the heights of the intestinal villi was observed (*P* < 0.01 or *P* < 0.05), compared with the other groups. Indicating that immunizing chickens with *B.S.* or *B.S.-HA* may effectively enhance nutrient absorption and growth rates. Interestingly, lymphoid tissue in the ileum increased significantly in the *B.S.-HA* group (*P* < 0.01 or *P* < 0.05), compared with the PBS group after 7 week immunization. This result indicates that *B.S.-HA* enhances the proliferation and development of intestinal lymphoid tissue.

### The levels of TLRs and cytokines

**Figure 5:**
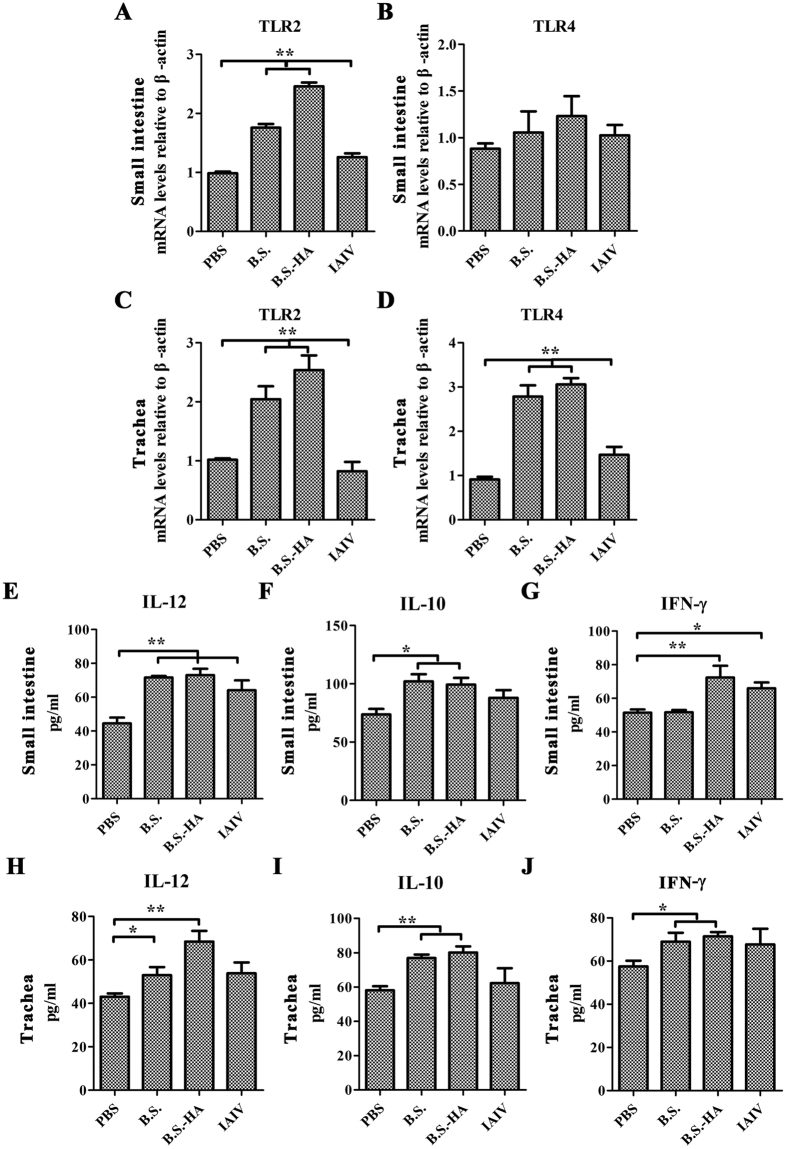
The expression levels of Toll-like receptors (TLRs) and cytokines. TLR2 and TLR4 mRNA expression in the small intestine (**A,B**) and trachea (**C,D**) were measured at day 3 after the second immunization. IL-12, IL-10, and IFN-γ cytokines expression levels in the small intestine (**E–G**) and trachea (**H–J**) suspensions were measured at day 3 after the second immunization. Data shown are the means ± s.d. of six samples. *P < 0.05; **P < 0.01. The error bars represent standard deviations.

These results suggest that *B.S.-HA* enhances the immune response in chickens. To determine whether immunization induces a mucosal cell-mediated immune response. TLR2 and TLR4 expression was measured in the trachea and small intestine, and IL-12, IL-10, and interferon (IFN-)-γ secretion levels in the tracheal and small intestinal suspensions were measured at 3 days after the second immunization (Fig. 5). TLR2 and TLR4 mRNA expression levels in the small intestine and trachea of chickens immunized with *B.S.-HA* were upregulated significantly compared with PBS and inactivated avian influenza virus (IAIV) (*P* < 0.05 or *P* < 0.01), besides the TLR4 mRNA expression in the small intestine. The levels of IL-12, IL-10, and IFN-γ secreted in the small intestine and trachea of chickens immunized with *B.S.-HA* and IAIV were upregulated significantly compared with those of chickens immunized with PBS (*P* < 0.05 or *P* < 0.01).

### The proliferation of spleen lymphocytes *in vitro*

**Figure 6:**
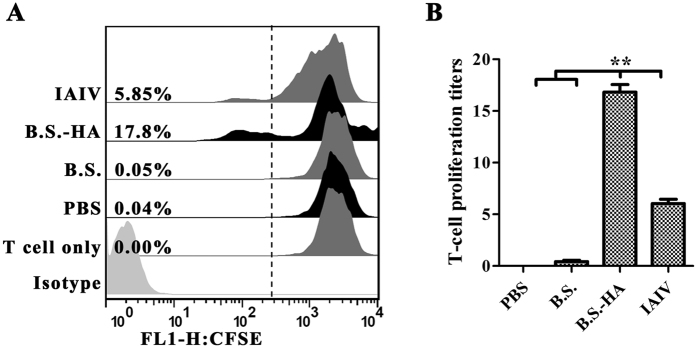
The proliferation of spleen lymphocytes *in vitro*. (**A**) HA-specific splenic lymphocyte proliferative reaction. Cells were cultured in a lymphocyte culture medium of 2 × 10^5^ cells per well in 24-well culture plates and stimulated by the purified recombinant HA protein (final concentration 10 μg/ml) for 72 h. Cell proliferation assays were detected by flow cytometry. Non-stimulated cells were used as negative controls; (**B**) The data is displayed in the histogram. Data shown are the means ± s.d. of three samples. *P < 0.05; **P < 0.01. The error bars represent standard deviations.

To examine the proliferative response of splenic lymphocytes primed with the HA protein *in vitro*, splenic lymphocytes were isolated from chickens 7 weeks after immunization. After the splenic lymphocytes were stimulated by HA protein *in vitro*, their proliferative index was increased markedly after immunization with *B.S.-HA (P* < 0.01) (Fig. 6).

### The changes of AIV-specific immune responses

**Figure 7:**
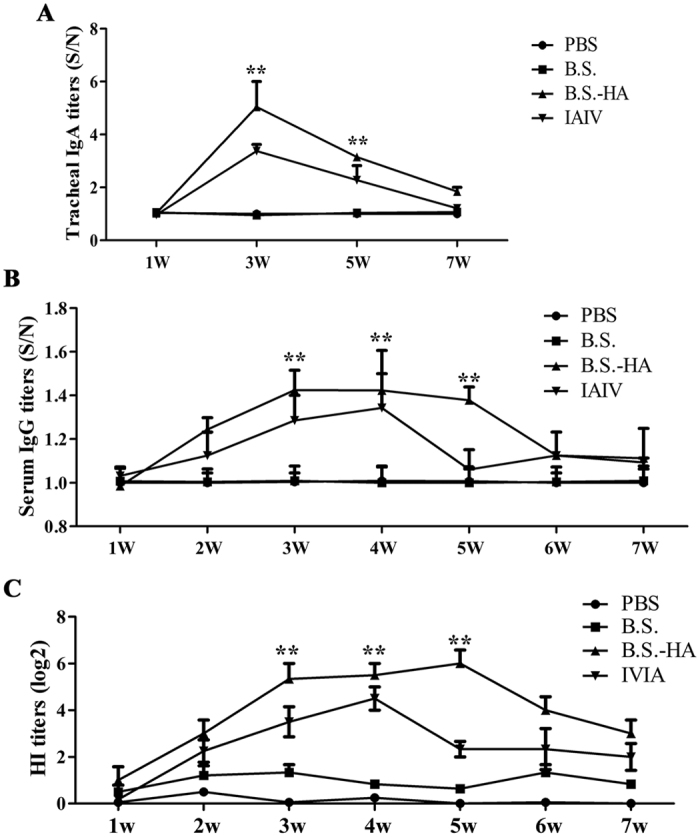
*B.S.-HA* enhanced mucosal and systemic immune responses after oral immunization. (**A**) The trachea suspensions after 1, 3, 5, and 7 week immunization were analyzed specific IgA (S/N) by indirect ELISA. (**B**) Serum after 1-7 week immunization were analyzed specific IgG (S/N) by indirect ELISA. (**C**) HI antibodies in serum were analyzed by hemagglutination inhibition (HI) assays. Data shown are the means ± s.d. of six samples. *P < 0.05; **P < 0.01. The error bars represent standard deviations.

Specific IgA antibody levels were measured 1, 3, 5, and 7 weeks after the first immunization. High levels of mucosal specific IgA antibody were observed in the tracheal suspensions and increased significantly 3 and 5 weeks after oral immunization with *B.S.-HA* (P < 0.01; Fig. 7A) compared with immunized with PBS or *B.S*. The level of mucosal specific IgA antibody was also increased in the group immunized with IAIV.

Specific immunity can be effectively promoted by cellular and innate immunities. Serum IgG antibody levels were detected 1–7 weeks after the first immunization. Serum antibody levels increased (P < 0.01; Fig. 7B), peaking at 3–5 weeks after immunization with *B.S.-HA* compared with immunization with PBS or *B.S*. In addition, the IAIV induced higher levels of IgG antibodies at weeks 3 and 4.

The serum antibody titer was detected by the HI assay (Fig. 7C). Serum titers from group that immunized with *B.S.-HA* increased at 3 weeks and peaked at 5 weeks after the first immunization. In addition, IAIV induced a higher titer 4 weeks after the first immunization. These results show that oral administration of *B.S.-HA* effectively induced mucosal immunity, a systemic immune response, and resistance to the infection by AIV to a certain extent.

## Discussion

*B. subtilis* is a well-characterized spore-forming bacterium widely used to express heterologous proteins and deliver antigens^8^,^19^,^20^,^21^. *B. subtilis* is a probiotic microorganism in broilers and laying hens^22^,^23^,^24^. Previous studies have indicated that dietary supplementation of *B. subtilis* exerts a beneficial effect on intestinal microbiota and gut morphology thereby enhancing growth performance and improving the feed conversion ratio in animals^18^. These effects of *B. subtilis* are probably due to its ability to produce amylase, protease, lipase and amino acids^25^, which could increase the efficiency of digestion and absorption of nutrients. Researchers generally agree that the improved growth performance observed in response to probiotics is associated with healthy modulation of the gut community composition^26^. In addition, *B. subtilis* has been used as an adjuvant to stimulate the immune response^27^.

DCs are the most potent professional antigen-presenting cells and play a crucial role in linking of innate and adaptive immunities^28^. *B. subtilis* recruits more DCs, migrates to mesenteric lymph nodes, and induces an immune response. Our previous experiments showed that *B. subtilis* increases the MHC II phenotype in pig BM-DCs *in vitro* and stimulates the uptake ability of DCs^29^. In this study, recombinant *B. subtilis* was sampled by BM-DCs *in vitro*, and the percentage of BM-DCs containing *B. subtilis* increased with the quantity of bacteria used. Interestingly, the quantity of the recombinant bacteria in DCs decreased as the incubation time extended. It reported that recombinant HA proteins from H5N1 influenza viruses are capable of activating mouse DCs activation and suppressing endocytosis^30^. After the *B.S.-HA* sampled by chicken BM-DCs a period of time, the exposed HA protein inhibited the endocytosis of chicken BM-DCs.

TLRs are required to activate innate and adaptive immune responses^31^,^32^. *B. subtilis* is recognized by TLR2 or TLR4 and augments mucosal and systemic responses to intranasal antigens in mice^33^,^34^. *B. subtilis* could activate the nuclear factor kappa B pathway by binding to TLRs and induced production of primary T helper type 1 (Th1) cytokines such as IL-1, IFN-γ, and IL-12^35^. Our results show that *B.S.-HA* upregulate secretion of IFN-γ and IL-12 in the chicken small intestine and trachea. These cytokines further activated T cells, consequently enhanced immune response. The IL-10 as Th2-related cytokine, which could be induced by *B.S.-HA* in chickens small intestine and trachea, can promote the maturation and activation of B cells, further increase the production of antigen specific IgA and IgG antibodies^36^.

According to the “common mucosal immune system” theory, antigen-sensitized precursor B and T lymphocytes generated at one mucosal site (such as the gut) can be detected at anatomically remote and functionally distinct compartments (such as the respiratory mucosa)^37^. In addition, avian influenza H5N1 has been detected in the nasal cavity mucosa, lungs, cloaca, and serum of aerosol-infected chickens^38^. Secretory IgA antibodies are the major contributors to humoral mucosal immunity against an influenza viral infection^39^. Our results show that oral administration of *B.S.-HA* increased the specific IgA antibody level in tracheal suspensions. The mucosal vaccine induced mucosal and systemic immune responses. Systemic immunity plays an important role in preventing the H5N1 virus. In this study, serum specific IgG and HI antibody titers were boosted by oral immunization of *B.S.-HA*, indicating that the recombinant *B. subtilis* effectively stimulated the systemic immune response.

## Conclusion

This study indicated that the recombinant *B.S.-HA* upregulated the MHC II phenotype expression in BM-DCs to sample *B.S.-HA*, and induced immune responses. Immunized chickens with *B.S.-HA* significantly elevated specific IgA titers in trachea, IgG and HI antibody titers in serum.

## Additional Information

**How to cite this article**: Mou, C. *et al*. Immune Responses Induced by Recombinant *Bacillus Subtilis* Expressing the Hemagglutinin Protein of H5N1 in chickens. *Sci. Rep.* **6**, 38403; doi: 10.1038/srep38403 (2016).

**Publisher's note:** Springer Nature remains neutral with regard to jurisdictional claims in published maps and institutional affiliations.

## Supplementary Material

Supplementary Material

## Figures and Tables

**Table 1:** Oligonucleotides list.

Name	Sequence (5′-3′)
F1	CGGATCCACCATGGAAACAACTCG
R1	GCCCGGGTTAAATGCAAATTCTGC
TLR2 F	GATTGTGGACAACATCATTGACTC
TLR2 R	AACGCTGCTTTCAAGTTTTCCC
TLR4 F	TGACCTACCCATCGGACACT
TLR4 R	CTCAGGGCATCAAGGTCTCC
β-actin F	ATGAAGCCCAGAGCAAAAGA
β-actin R	GGGGTGTTGAAGGTCTCAAA
