# Supplementary material for: Immune Responses Induced by Recombinant Bacillus Subtilis Expressing the Hemagglutinin Protein of H5N1 in chickens
Source: Sci Rep. 2016 Dec 16;6:38403. doi: 10.1038/srep38403 (PMC5159790; doi:10.1038/srep38403)
Supplement: Supplementary Material [file srep38403-s1.doc]

**Immune Responses Induced by** **Recombinant *Bacillus Subtilis***

**Expressing the Hemagglutinin Protein of H5N1 in chickens**

Chunxiao Mou^1^, College of Veterinary Medicine, Nanjing Agricultural University, Weigang 1, Nanjing, Jiangsu, 210095, PR China. Email: ytqxmcx@163.com,

Liqi Zhu^2^, College of Veterinary Medicine, Nanjing Agricultural University, Weigang 1, Nanjing, Jiangsu, 210095, PR China. Email: zhuliqio2@163.com,

Jingjing Yang^3^, College of Veterinary Medicine, Nanjing Agricultural University, Weigang 1, Nanjing, Jiangsu, 210095, PR China. Email: july0108@163.com,

Wenwen Xu^4^, College of Veterinary Medicine, Nanjing Agricultural University, Weigang 1, Nanjing, Jiangsu, 210095, PR China. Email: xww623@126.com,

Xiaoying Cheng^5^, College of Veterinary Medicine, Nanjing Agricultural University, Weigang 1, Nanjing, Jiangsu, 210095, PR China. Email: 961178447@qq.com,

Corresponding author: Qian Yang, College of Veterinary Medicine, Nanjing Agricultural University, Weigang 1, Nanjing, Jiangsu, 210095, PR China. Email: zxbyq@njau.edu.cn.


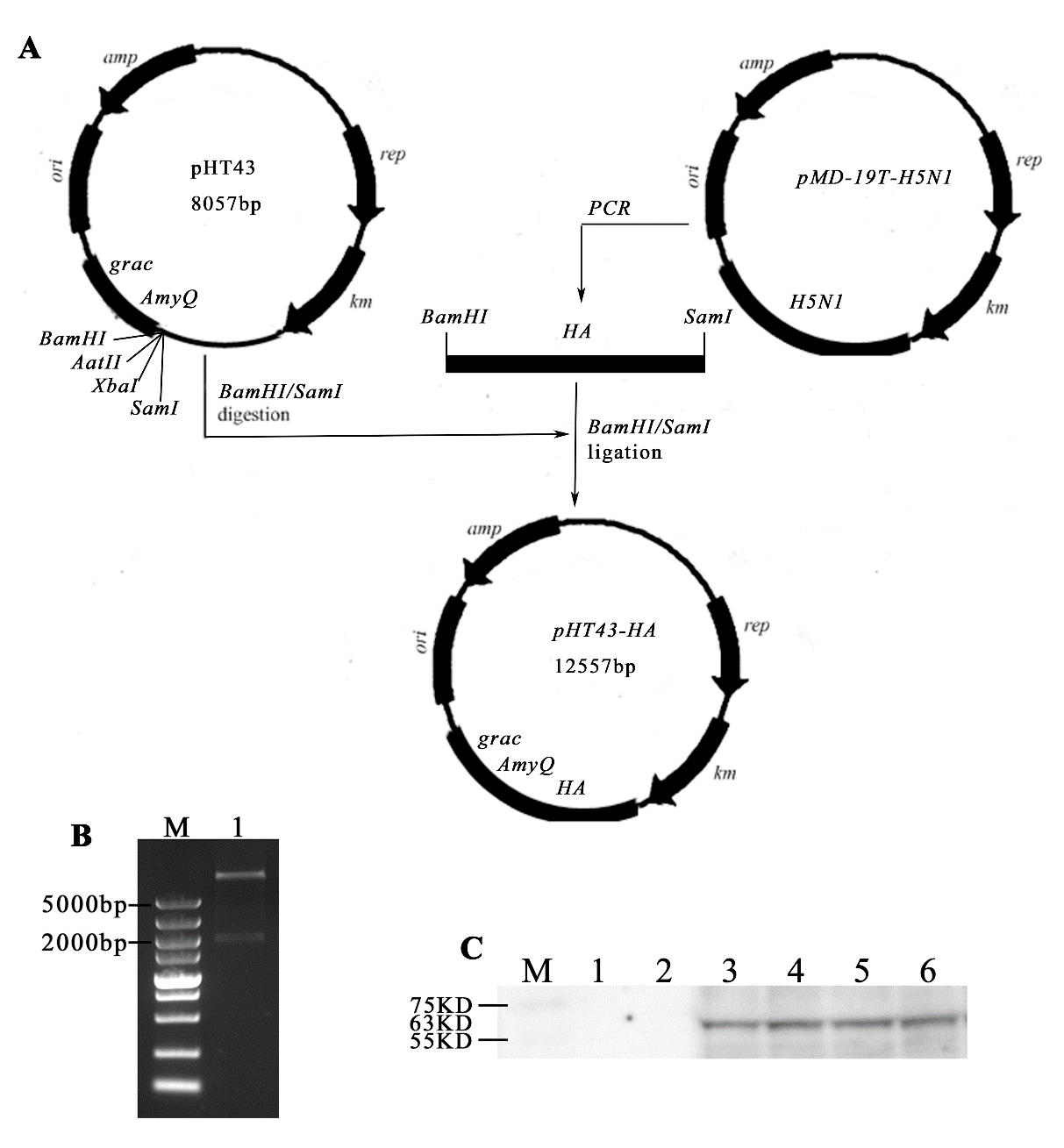


**Supplementary material fig 1.** The constitution of *pHT43-HA* plasmid and the detection of HA protein. (**A**) HA fragment was amplified from *pMD-19T-HA* plasmid, inserted into vector *pHT43* plasmid by T4 DNA ligase to generate the vector named *pHT43-HA*. (**B**) Identification of the *pHT43-HA* plasmid with restriction enzymes. Recombinant *pHT43-HA* plasmid lane 1, digested with *BamH*I and *Sma*I the HA gene (2000 bp) and the backbone of the plasmid; M, DL 5000. (**C**) Western blotting using anti-HA; lane 1 and 2, *B. subtilis* WB800N; lane 3, 4, 5, 6, *B.S.-HA*. Protein bands of approximately 63 KD, which correspond to the expected size of HA protein, were detected.
